# Removal of Fe^2+^ and Mn^2+^ from Polluted Groundwater by Insoluble Humic Acid/Tourmaline Composite Particles

**DOI:** 10.3390/ma15093130

**Published:** 2022-04-26

**Authors:** Ling Liu, Tianyi Zhang, Xiaowan Yu, Vitumbiko Mkandawire, Jiadi Ma, Xilin Li

**Affiliations:** 1 School of Civil Engineering, Liaoning Technical University, Fuxin 123000, China; liuling@lntu.edu.cn (L.L.); zty18342846242@163.com (T.Z.); yuxiaowan5158@163.com (X.Y.); vitumbikomkandawire@rocketmail.com (V.M.); majiadi0924@163.com (J.M.); 2 Information Industry Electronics Eleventh Design and Research Institute Technology Engineering Co., Ltd., Dalian Branch, Dalian 116000, China; 3 Water Services Association of Malawi, Tikwere House, City Center, Private Bag 390, Lilongwe 207213, Malawi

**Keywords:** insoluble humic acid, tourmaline, iron ion (Fe^2+^), manganese ion (Mn^2+^), adsorption kinetics, isothermal adsorption, removal mechanism

## Abstract

Insoluble humic acid/tourmaline composite particles (IHA/TM) were prepared by combining inorganic tourmaline (TM) with the natural organic polymer humic acid (HA) and carbonizing them at 330 °C to study the removal characteristics and mechanism of Fe^2+^ and Mn^2+^. The results showed that the optimal ratio of TM to IHA is 2:3. When the temperature of the IHA/TM composite particles was 35 °C and the pH was 6, the adsorption of Fe^2+^ and Mn^2+^ by IHA/TM reached equilibrium at 240 min. The optimum dose of the adsorbent was 10 g/L, and the equilibrium adsorption capacities of Fe^2+^ and Mn^2+^ were 5.645 mg/g and 3.574 mg/g, respectively. The process of IHA/TM adsorption of Fe^2+^ and Mn^2+^ in water was spontaneous, endothermic and sustainable, and cooling was not conducive to adsorption. The pseudo-second order kinetic equation can well reflect the adsorption mechanism of IHA/TM on Fe^2+^ and Mn^2+^, and the Langmuir adsorption model better describes the isothermal adsorption behaviour. The material characterisation and adsorption experiments indicate that surface coordination and chemical precipitation are the main mechanisms of Fe^2+^ and Mn^2+^ removal by IHA/TM.

## 1. Introduction

Iron and manganese are the fourth and second most abundant metal elements in the Earth’s crust and are usually present in groundwater in the form of divalent ions (Fe^2+^ and Mn^2+^) [1]. There are two main sources of these metals: geological sources and anthropogenic sources. Geological sources include minerals containing Fe and Mn, which dissolve when water penetrates soil and rocks and preserves them in an aqueous solution. Anthropogenic sources include industrial wastewater, landfill leakage, acid mine wastewater, etc., all of which result in high concentrations of Fe^2+^ and Mn^2+^ in groundwater [2]. There are serious iron and manganese pollution problems in groundwater in mining areas of northeast, northwest and north China due to mining and disorderly discharge of industrial wastewater. The concentrations of Fe^2+^ and Mn^2+^ in polluted groundwater in Shuangyashan mining area of Heilongjiang and Jilin Tonghua mining area in Northeast China can reach up to 60.00 mg/L and 14.17 mg/L [3]; the Fe^2+^ content in the polluted water of the Malan coal mine in North China reaches 102.90 mg/L, the Mn^2+^ content is 8.10 mg/L, and the pH is 4.83 [4]; the content of Mn^2+^ in the weakly acidic mine drainage of the Enhong coal mine in Northwest China is as high as 32 mg/L, and the iron ion content is as high as several hundred mg/L [5]. High concentrations of Fe^2+^ and Mn^2+^ exist in groundwater, which will not only cause serious color problems, but also damage the local ecological environment. Long-term excessive intake of iron and manganese-contaminated water caused by water shortage in mining areas may cause chronic poisoning and affect health [6]. How to remove the pollution of high concentrations of Fe^2+^ and Mn^2+^ in groundwater has always been the concern of the majority of environmental workers [7].

Domestic and foreign treatment methods for removing Fe^2+^ and Mn^2+^ from groundwater mainly include filtration, oxidation, precipitation, biological treatment, adsorption method and membrane technology [8,9]. Among them, the adsorption method has been proved to be a technology with low cost, good effect, high efficiency, and is easy to operate [10]. Selecting and preparing the adsorption material for Fe and Mn removal are key to research and application. Geremias et al. used coal mine tailings as adsorbents and successfully adsorbed iron and manganese ions in acidic mine drainage [11]. Apkar’yan et al. prepared an environmentally friendly glass-ceramic particle material based on broken glass, red clay and organic additives, which can effectively remove iron and manganese ions from groundwater [12]. Aziz et al. discovered the great potential of zeolite as a good adsorbent for heavy metal ions by exploring the adsorption capacity of activated clinoptilolite for iron and manganese ions in acid wastewater from palm oil mills [13]. In addition, some low-cost and easily available waste materials such as rice husk ash, citrus peel, and natural minerals such as hydroxyapatite have also become the current application hotspots [14,15]. However, the existing adsorbents for removing iron and manganese ions are still insufficient in terms of cost and adsorption effect, especially for groundwater contaminated with high concentrations of iron and manganese ions. The ion adsorbent has become the key to research and application, which has also become the focus of our research.

Tourmaline (TM) is a complex cyclic silicate mineral with high mechanical and chemical stability. It can continuously release negative ions, has spontaneous polarity, and does not pollute the environment. TM is a relatively environmentally friendly material with spontaneous electrode characteristics. In 1989, Kubo et al. found that tourmaline has the characteristics of a spontaneous electrode and first proposed the existence of an electrostatic field on the surface of tourmaline [16]. Subsequently, many scholars have conducted research on the adsorption ability and removal of heavy metal ions by tourmaline [17]. However, TM has the disadvantages of high agglomeration and difficult removal from water due to its large surface energy [18]. Currently, the use of surface modification or preparation of composite materials to reduce agglomeration and improve adsorption performance has become a common research topic in the application of TM [19]. Chen et al. synthesized bentonite and tourmaline montmorillonite composites (TMMs) by a vacuum sintering method that can better adsorb Pb (II) in water [20]. Liao et al. reported that silver-loaded polyacrylic acid-modified tourmaline composites could efficiently remove methylene blue and Cu (II) ions from water by catalytic degradation [21]. Studies have shown that the compounds used for surface modification and composite adsorbents have an important characteristic in common: strong metal complexing abilities. Humic acid (HA) is such a material. HA, a polymeric organic material that widely exists in nature, contains carboxyl, hydroxyl, carbonyl and other active groups that can adsorb onto heavy metals and undergo complexation and redox reactions with heavy metals in the environment [22,23]. However, as an adsorbent, humic acid has low mechanical strength, is difficult to separate from water and has poor biological stability, so it is still not directly used as an adsorbent; thus, the development of insoluble humic acid (IHA) has become an important research topic. Zhao et al. made insoluble humic acid and used it to remove Mn^2+^ from aqueous solution. At pH = 5.6 and at 25 °C, the rate of Mn^2+^ removal by insoluble humic acid was 92% [24]. Wei et al. prepared chitosan-crosslinked insoluble humic acid (CS @ IHA) by a dropping ball method, and this material had a high adsorption capacity for Pb (II) in water [25]. The combination of IHA and TM can not only change the surface properties and environmental behaviours of TM but also affect the adsorption effect of pollutants.

Based on this behaviour, a new composite adsorbent, insoluble humic acid/tourmaline (IHA/TM), was prepared by making full use of the respective advantages of HA and TM. The thermodynamic and kinetic behaviours of Fe^2+^ and Mn^2+^ were analysed, the key parameters of adsorption and adsorption capacity were determined, and the microscopic removal mechanism was discussed to provide a scientific basis and technical background for the practical application of IHA/TM in the treatment of groundwater containing Fe^2+^ and Mn^2+^.

## 2. Materials and Methods

### 2.1. Preparation of IHA/TM Composite Particles and Determination of Their Optimum Ratio

TM is mainly composed of Schorl. It had a particle size of 300 mesh, and its chemical formula was NaFe_3_Al_6_(BO_3_)_3_SiO_6_O_18_(OH)_4_. The chemical composition of tourmaline was determined by X-ray fluorescence (XRF) as follows: SiO_2_ 36.28%, Al_2_O_3_ 22.86%, Fe_2_O_3_ 19.41%, B_2_O_3_ 7.81%, MgO 4.66%, CO_2_ 3.23%, CaO 2.30%, Na_2_O 1.77% and other 1.68%.

Preparation of IHA: HA was dissolved in 0.1 mol/L NaOH solution and undissolved impurities were discarded. The pH of the HA-containing solution was adjusted to 1.2 ± 0.2 with 6.0 mol/L HCl and placed in a water bath for heating at 65 ± 5 °C for 2 h. The precipitated HA was dried in an oven at 45 ± 5 °C to produce IHA.

Preparation of IHA/TM composite particles occurred as follows. The pretreated IHA was dissolved in 0.1 mol/L NaOH solution, and then controlled TM was mixed with IHA at an optimal mixing ratio. The pH was adjusted to 6.0, the mixture was shaken (25 °C, 150 r/min) for 12 h and centrifuged. The precipitate was washed three times with deionized water and then dried in an oven at 65 ± 5 °C for 24 h [26]. After grinding and screening, the solid was mixed with etherified starch (10/1, (*v/v*)) which was dextrinated at 95 °C to make adsorbent pellets with 3–5 mm particles, which were calcined in a muffle furnace at 300 °C for 1 h to form IHA/TM composite particles.

The optimum ratio of IHA and TM was determined. Based on the characteristics of typical groundwater high in iron and manganese in Fuxin, Liaoning Province, China, the Fe^2+^ content of the water sample was set at 25 mg/L, and the Mn^2+^ content was set at 10 mg/L. The mixing ratios of IHA and TM were controlled at 1:3, 2:3, 1:1, 3:2 and 3:1 in the preparation of the composite particles, and 1.2 g of composite particles was placed in a 100 mL water sample. After shaking in a shaker (25 °C, 150 r/min) for different times, the concentration of Fe^2+^ and Mn^2+^ and the pH value of the solution were measured.

### 2.2. Material Characterisation

Changes in surface morphology, particle size and agglomeration were analysed using a JEOL JSM-7500F scanning electron microscope (SEM, Tokyo, Japan). Elemental and content analyses of the materials were performed with an FYFS-2002E energy dispersive spectrometer (EDS). An XRD-6100 X-ray diffractometer (XRD, Shimadzu, Japan) was used for sample phase analysis. FTIR studies were performed with an IRPrestige-21 Fourier transform infrared spectrometer to characterise molecular structures, chemical bonding and functional group changes in adsorption materials.

### 2.3. Static Adsorption Test

#### 2.3.1. Effect of IHA/TM Dose on Adsorption

The experimental study simulated the Xinqiu mining area in Liaoning, China. In this area, due to mining and industrial wastewater discharge, the groundwater is seriously polluted by iron and manganese ions. The maximum concentrations of Fe^2+^ and Mn^2+^ were 20.5–25.0 mg/L and 5.9–9.8 mg/L, respectively, and the pH was 5.8–6.1. A 100 mL of water sample with Fe^2+^ content 25 mg/L and Mn^2+^ content 10 mg/L was taken, pH adjusted to 6.0 with 0.1mol/L HCl and NaOH solution, and dosage of IHA/TM controlled to be 0.4–1.4 g. IHA/TM was weighed accurately and added to the water sample, shaken at 150 r/min for a fixed time at 25 °C, and after centrifugation, the concentrations of Fe^2+^ and Mn^2+^ were measured.

The amount of Fe^2+^ and Mn^2+^ adsorbed on IHA/TM per unit mass and removal rate *R* (%) were calculated using Equations (1) and (2), respectively [27].

(1) $$q_{e}=\frac{\;(C_{0}-C_{e})\;V}{m}$$

(2) $$R=\frac{C_{0}-C_{e}}{C_{0}}\times 100\%$$

where *q*_e_ is the adsorption capacity of IHA/TM composite particles at equilibrium, mg/g; *C*_0_ is the initial concentration of groundwater containing Fe^2+^ and Mn^2^, mg/L; *C*_e_ is the solution concentration when the adsorption reaches equilibrium, mg/L; *V* is the solution volume, L; and *m* is the adsorbent mass, g.

#### 2.3.2. Effect of Initial pH on Adsorption

To determine the effect of the initial pH on the adsorption performance of IHA/TM, 1.0 g of IHA/TM composite particles was added to 100 mL of water with an Fe^2+^ content of 25 mg/L and Mn^2+^ content of 10 mg/L, and the pH was adjusted to 2.0–9.0. The samples were shaken at 150 r/min for 240 min at 25 °C, and the concentrations of Fe^2+^ and Mn^2+^ were measured after centrifugation.

#### 2.3.3. Adsorption Isotherm Experiment

Different initial concentrations of Fe^2+^ and Mn^2+^ solutions were separated into 100 mL aliquots in 250 mL conical flasks, and the IHA/TM composite particles were stored in 1.0 g samples at pH 6. The filtrate was shaken at 150 r/min for 240 min at different temperatures to determine the Fe^2+^ and Mn^2+^ concentrations.

#### 2.3.4. Adsorption Kinetics

Fe^2+^ and Mn^2+^ solutions (100 mL) with different initial concentrations were placed in 250 mL conical flasks. The dosage of IHA/TM composite particles was kept at 1.0 g at a pH of 6.0 and temperature of 25 °C. The concentrations of Fe^2+^ and Mn^2+^ in the filtrate were measured after different reaction times.

#### 2.3.5. Regeneration Test

To evaluate the reusability of the IHA/TM composite particles, the IHA/TM composite particles after adsorption saturation were centrifugally filtered, the unabsorbed Fe^2+^ and Mn^2+^ ions were washed away, mixed with 0.1 mol/L HNO_3_ solution, and shaken at 25 °C for 240 min washed with deionized water until neutral, and dried at 105 °C. The adsorption and desorption experiments were repeated 5 times using the same batch of IHA/TM composite particles, and the removal rates of Fe^2+^ and Mn^2+^ by IHA/TM after each desorption were calculated.

## 3. Results and Discussion

### 3.1. Optimal Ratio of IHA and TM

**Figure 1:**
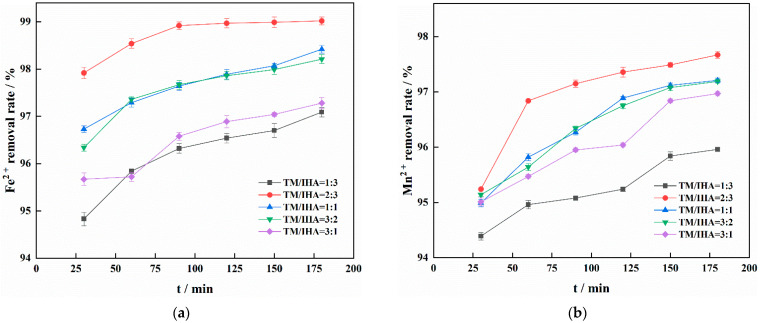
Effect of different mixing ratios on the removal of Fe^2+^ (**a**) and Mn^2+^ (**b**).

As shown in Figure 1, the mixing ratio of IHA and TM greatly influences the rate of Fe^2+^ and Mn^2+^ removal. IHA can improve the dispersion performance of TM and reduce its agglomeration, and its surface has many active groups (such as -COO-, -COOH, and -OH) that can transfer electrons and improve the adsorption capacity [28]. However, these IHA functional groups also occupy the active sites of TM, reducing the adsorption performance of the adsorbent [29]. Therefore, there is an optimal TM:IHA ratio. When TM/IHA = 2:3, the rates of Fe^2+^ and Mn^2+^ removal were 99.02% and 97.67%, respectively. In this study, a composite material with TM/IHA = 2:3 was selected as the experimental adsorbent to explore the adsorption characteristics of the adsorbent for Fe^2+^ and Mn^2+^.

### 3.2. Effect of IHA/TM Dose on the Adsorption of Fe^2+^ and Mn^2+^

**Figure 2:**
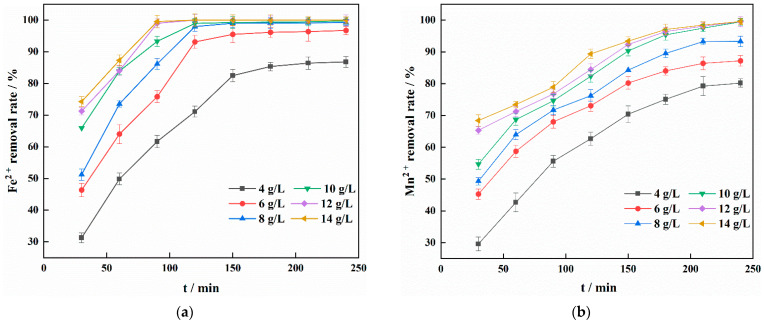
Effect of different doses on the removal of Fe^2+^ (**a**) and Mn^2+^ (**b**).

Figure 2 shows that with increasing IHA/TM composite particle dose, the rates of Fe^2+^ and Mn^2+^ removal first increased and then stabilised. This stabilisation is due to the increase in IHA/TM, which provides many surface active sites, increases the contact probability of IHA/TM with Fe^2+^ and Mn^2+^, and increases the rate of Fe^2+^ and Mn^2+^ removal. When the dose was 10 g/L, the rates of Fe^2+^ and Mn^2+^ removal were 99.75% and 99.45%, respectively. The rates of Fe^2+^ and Mn^2+^ removal remained mostly stable as the dose was continually increased. The dose of IHA/TM was selected as 10 g/L by comprehensive comparison.

### 3.3. Effect of Initial pH on Adsorption of Fe^2+^ and Mn^2+^

**Figure 3:**
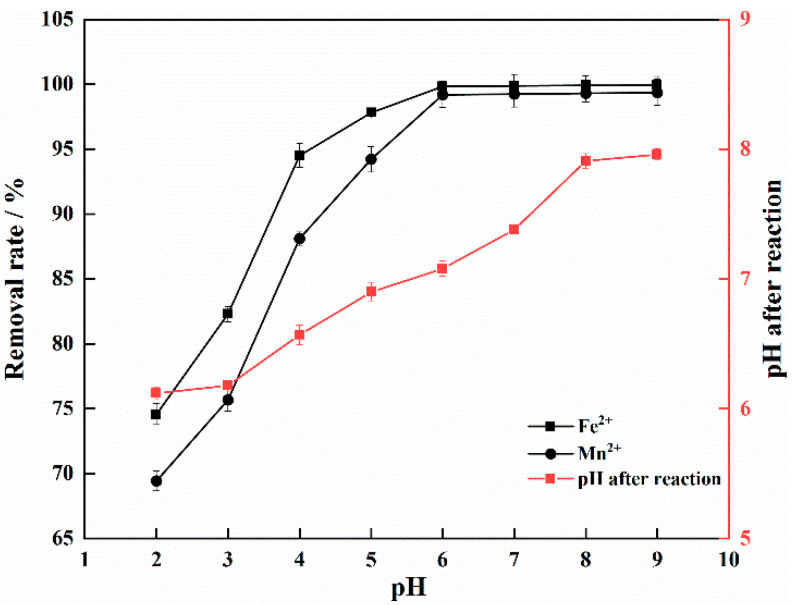
Effect of pH on the removal efficiency.

Studies have shown that the pH of the solution greatly influences the complexation reaction of TM, IHA and metal, and their complexing ability and stability increase with increasing pH [30]. Figure 3 shows that when the pH was 2–6, the rates of Fe^2+^ and Mn^2+^ removal increased significantly with increasing pH. When pH > 6, the rate of Fe^2+^ and Mn^2+^ removal tended to stabilise because the pH of the solution system not only affects the surface charge, ionisation degree and type of adsorbent, but also determines the forms of Fe^2+^ and Mn^2+^ present in the solution, resulting in a large difference in the performance of the adsorbent at different pH values [31].

When pH < 4, the high concentration of H^+^ competitively adsorbed onto the surface, blocking Fe^2+^ and Mn^2+^, and it more easily reacted with the active groups on the surface of the IHA/TM so that metal ions easily dissociated from the complex. When the pH was 4–6, the rate of Fe^2+^ and Mn^2+^ removal continued to increase, but the growth rate slowed slightly. The adsorption of H^+^ on the adsorbent was reduced, while H^+^ dissociated from functional groups such as carboxyl and hydroxyl groups as they were deprotonated. Therefore, the negative charge on the IHA/TM surface increased, and the adsorption sites increased [32]. Under acidic conditions, some of the Fe^2+^ combined with OH^−^ to form Fe(OH)^+^, the surface charge decreased, the electrostatic repulsion between the surface protonated adsorbent weakened, and Fe^2+^ was more easily adsorbed. In addition, the active functional groups of IHA tended to form complexes with iron and manganese ions at a high pH, which is more conducive to the formation of humic acid-iron-manganese complexes, thereby reducing Fe^2+^ and Mn^2+^ in the solution [33]. However, under neutral and basic conditions, Fe^2+^ and Mn^2+^ were precipitated as Fe(OH)_2_ and Mn(OH)_2_, nearly completing their removal.

Due to the good buffer performance of the TM in the adsorbent, the pH of the solution tended to be neutral after the reaction reached equilibrium. This phenomenon was due to the spontaneous polarity of TM (chemical Equations (3)–(5)). This electrode allowed the surrounding air to become weakly electrolyzed, H^+^ obtained electrons, and OH^−^ combined with H_2_O to generate negative ions. The performance was also related to the hydroxyl (-OH) crystal structure and the numerous atomic bonds on the surface. TM may be a very good adsorbent for the remediation of Fe^2+^ and Mn^2+^ contaminated water because it can be applied over a wide pH range [34].

(3) $$H_{2}O\to H^{+}+{\mathrm{OH}}^{-}$$

(4) $${2H}^{+}+2e^{-}\to H_{2}↑$$

(5) $${\mathrm{OH}}^{-}+{\mathrm{nH}}_{2}O\to {\mathrm{OH}}^{-}{\left(H_{2}O\right)}_{n}$$

The removal of Fe^2+^ and Mn^2+^ by adsorption onto IHA/TM is more successful under weakly acidic conditions. In this study, an initial pH of 6 was chosen; at this pH, Fe^2+^ and Mn^2+^ did not precipitate, and the rates of Fe^2+^ and Mn^2+^ removal were 99.84% and 99.20%, respectively.

### 3.4. Isothermal Adsorption Analysis

**Figure 4:**
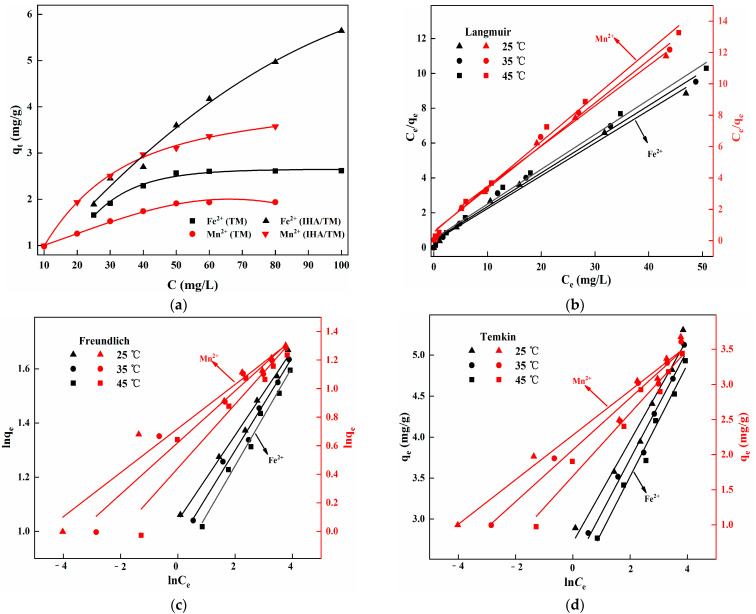
Isothermal adsorption of Fe^2+^ and Mn^2+^ onto TM and IHA/TM (**a**); equation fitting curve for the Langmuir model (**b**), Freundlich model (**c**) and Temkin model (**d**).

Figure 4a shows that at 35 °C, the *q*_e_ values of TM for Fe^2+^ and Mn^2+^ adsorption were 2.615 mg/g and 1.937 mg/g, respectively, while the equilibrium adsorption capacities of IHA/TM for Fe^2+^ and Mn^2+^ were 5.645 mg/g and 3.574 mg/g, respectively. The capacity of IHA/TM for iron and manganese removal was 2.16 and 1.85 times higher than that of TM, indicating that the removal capacity of IHA/TM for Fe^2+^ and Mn^2+^ was better than that of TM.

To explore the adsorption behaviour of Fe^2+^ and Mn^2+^ on IHA/TM, the Langmuir, Freundlich and Temkin models were used to fit the isothermal adsorption data (Figure 4b–d).

Langmuir isothermal adsorption equation [35]:

(6) $$\frac{C_{e}}{q_{e}}=\frac{1}{\left(K_{L}q_{m}\right)}+\frac{C_{e}}{q_{m}}$$

Freundlich isothermal adsorption equation:

(7) $$\mathrm{Ln}q_{e}=\mathrm{Ln}K_{F}+\frac{1}{n}\mathrm{Ln}C_{e}$$

Temkin isothermal adsorption equation:

(8) $$q_{e}=B\ln A+B\ln C_{e}$$

where *C*_e_ is the adsorption equilibrium solution concentration, mg/L; *q*_e_ and *q*_m_ are adsorption capacity at equilibrium and at saturation, respectively, mg/g; and *K*_L_, *K*_F_, *n*, *A* and *B* are all adsorption constants.

Linear fitting was performed using the Langmuir, Freundlich and Temkin models, and the relevant parameters are shown in Table 1. Based on *R*^2^ (>0.99), as shown in Table 1, the adsorption of Fe^2+^ and Mn^2+^ onto IHA/TM was in good agreement with the Langmuir isothermal adsorption equation. The results show that the adsorption of both Fe^2+^ and Mn^2+^ onto IHA/TM is monolayer adsorption, and the saturated adsorption capacity of IHA/TM for Fe^2+^ and Mn^2+^ increased with increasing temperature. For the dimensionless parameter separation factor *R*_L_, the expression *R*_L_ = 1/(1 + *K*_L_ *C*_0_) can be used to represent the adsorption properties of materials: the *R*_L_ value indicates that the adsorption type is unfavourable (*R*_L_ > 1), favourable (0 < *R*_L_ < 1), linear (*R_L_ =* 1) or irreversible (*R*_L_ = 0) [36]. The *R*_L_ of this experiment was 0–1, and the adsorption process of Fe^2+^ and Mn^2+^ onto IHA/TM was favourable.

### 3.5. Thermodynamic Analysis

To further determine the thermodynamic effects, chemical Equations (9) and (10) were used to estimate the thermodynamic parameters of the IHA/TM composite particles for the Fe^2+^ and Mn^2+^ adsorption processes. These parameters included the enthalpy change (Δ*H*), entropy change (Δ*S*) and Gibbs free energy change (Δ*G*).

(9) $$\Delta G=-RT\ln K_{d}$$

(10) $$\ln K_{d}=\frac{\Delta S}{R}-\frac{\Delta H}{RT}$$

(11) $$K_{d}=\frac{q_{e}}{C_{e}}$$

where *R* is the universal constant (8.314 J/(mol·*K*)), *T* is the absolute temperature (*K*), and *K*_d_ is the equilibrium constant calculated from Equation (11).

The enthalpy and entropy changes can be linearly fitted to the data based on the variation in the adsorption constants *K*_d_ with temperature. The results are given in Table 2. The negative values of Δ*G* for Fe^2+^ and Mn^2+^ indicate that the adsorption of IHA/TM is spontaneous and increases with increasing temperature. The positive values of Δ*H* for Fe^2+^ and Mn^2+^ indicate that the adsorption of IHA/TM is endothermic, and a higher temperature is more conducive to the reaction, which again verifies the conclusion of the Langmuir isothermal adsorption model. In addition, the positive value of Δ*S* indicates that the adsorption of Fe^2+^ and Mn^2+^ by the IHA/TM composite is accompanied by chemical reactions in which different types of ions are released into the liquid phase, indicating that the adsorption process is sustainable. Overall, the process of Fe^2+^ and Mn^2+^ adsorption onto IHA/TM is spontaneous, heat-absorbing and sustainable.

### 3.6. Adsorption Kinetic Analysis

**Figure 5:**
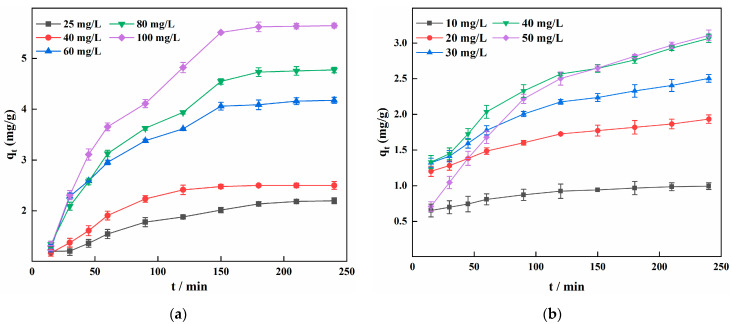
Effects of initial concentrations and contact time on the Fe^2+^ (**a**) and Mn^2+^ (**b**) adsorption amount of IHA/TM at 25 °C.

Figure 5 shows the variation in the adsorption capacity versus adsorption time for different initial concentrations of Fe^2+^ and Mn^2+^ on IHA/TM at a temperature of 25 °C, pH of 6, and a dose of 10 g/L. Two different removal stages can be observed; in the first stage, the adsorption capacity for Fe^2+^ and Mn^2+^ increases rapidly with increasing adsorption time, and then in the second stage, the adsorption capacity increases slowly until it remains almost constant and the reaction reaches dynamic equilibrium.

**Figure 6:**
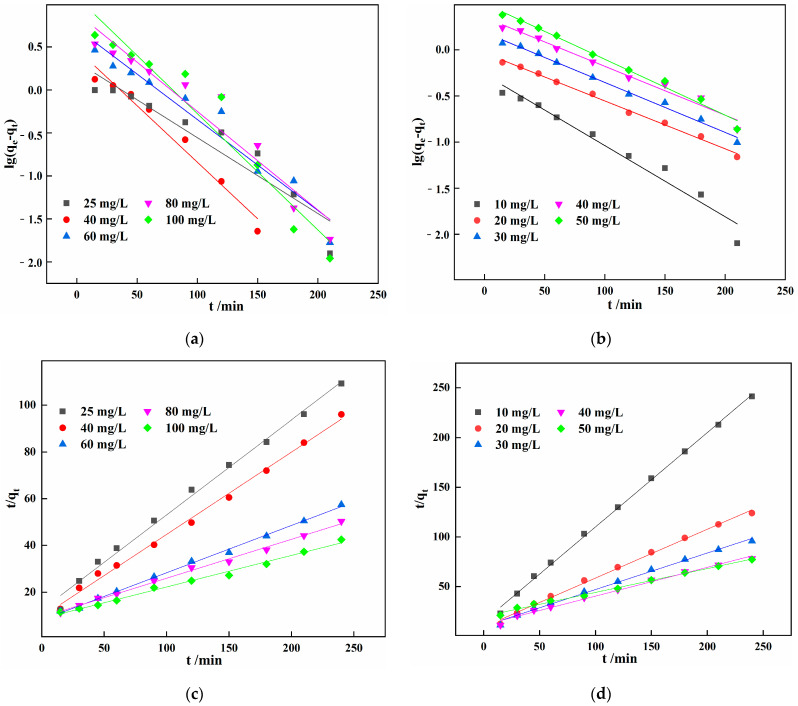
Quasi-first order kinetic equation fitting curve of IHA/TM adsorption of Fe^2+^ (**a**) and Mn^2+^ (**b**); quasi-second order kinetic equation fitting curve of IHA/TM adsorption of Fe^2+^ (**c**) and Mn^2+^ (**d**).

The Fe^2+^ and Mn^2+^ adsorption kinetics were fitted to quasi-first order (Equation (12)) and quasi-second order (Equation (13)) models to further elucidate the kinetic mechanism of Fe^2+^ and Mn^2+^ removal by IHA/TM [37] (Figure 6a–d).

(12) $$\mathrm{lg}(q_{e}-q_{t})=\mathrm{lg}q_{e}-\frac{K_{1}}{2.203}t$$

(13) $$\frac{t}{q_{t}}=\frac{1}{K_{2}\cdot q_{e}^{2}}+\frac{t}{q_{e}}$$

where *q*_e_ and *q*_t_ are the adsorption amount at equilibrium and *t* min, respectively, mg/g; *K*_1_ and *K*_2_ are the pseudo-first and second order kinetic rate constants, 1/min, mg/g/min.

The kinetic parameters of Fe^2+^ and Mn^2+^ adsorption by IHA/TM at different initial concentrations are given in Table 3. It can be seen from Table 3 that the *K*_1_ and *K*_2_ vary with the initial concentration regardless of Fe^2+^ or Mn^2+^, and the dispersion degree of kinetic constant *K*_2_ is larger than that of *K*_1_. With the increase in the initial concentration of Fe^2+^ and Mn^2+^, the kinetic rate constant *K*_2_ gradually decreased, which is consistent with the experimental results obtained by Nekouei et al. when the composite adsorbent CS-EDTA-mGO effectively adsorbed and removed Rhodamine B [38]. They believed that in the presence of a chemisorption reaction, the chemisorption rate was affected by the adsorbate concentration in the solution. Therefore, for the IHA/TM composite adsorbent, the complex chemical action occurs during the adsorption process, and the kinetic rate constant *K* is not independent of the initial concentrations of Fe^2+^ and Mn^2+^. This is different from the fact that the kinetic constant *K*, which is dominated by the physical adsorption process, is independent of the initial conditions [39]. It is very difficult to judge which kinetic model fits from the discreteness of the *K* value. Therefore, for the reaction process dominated by chemisorption, scholars still only judge the fitting kinetics from *R*^2^ and *q*_e_. The R^2^ values show that the fitting results of the adsorption kinetics of Fe^2+^ and Mn^2+^ onto IHA/TM are more consistent with the pseudo-second order kinetic equation (R^2^ > 0.99). The formation of chemical bonds is the main factor affecting pseudo-second order kinetics, indicating that adsorption mainly proceeds by chemisorption [40]. Surface adsorption, external liquid film diffusion and intraparticle diffusion explain the entire process of IHA/TM adsorption. Of course, when the adsorption reaction is chemical adsorption, it is worthwhile to further study the relationship between the adsorption kinetic rate constant *K*, the equilibrium adsorption capacity *q*_e_ and the initial concentration of pollutants, as well as the effect on the fitted kinetics.

To determine the diffusion mechanism, the Weber-Morris equation (chemical Equation (14)) was used for piecewise linear fitting of the experimental data.

(14) $$q_{t}=k_{p}t^{0.5}+C$$

where *q*_t_ is the adsorption amount at *t* min, mg/g; *k*_p_ is the diffusion rate constant, mg/g·min^−^^1/2^; and *C* is a constant related to the boundary layer thickness, mg/g.

**Figure 7:**
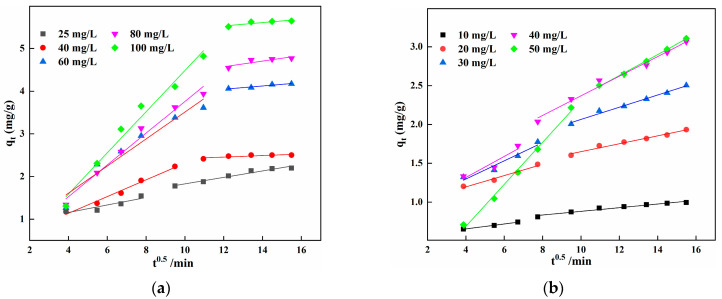
Fitting curve of the internal diffusion equation of Fe^2+^ (**a**) and Mn^2+^ (**b**).

In Figure 7, the fitted curves of *q*_t_ and *t*^0.5^ are divided into two stages for different concentrations of Fe^2+^ and Mn^2+^. In the initial stage, the slope of the straight line is greater, the adsorption rate is faster, and many vacant active sites on the IHA/TM are available for Fe^2+^ and Mn^2+^ adsorption. The adsorption reaction is mainly controlled by the ion exchange between Fe^2+^ and Mn^2+^, the functional groups on the surface of the IHA/TM adsorbent and the diffusion of the outer liquid film. In the second stage, the adsorption rate is lower, and as the binding sites are gradually occupied, a concentration gradient forms between the surface and the interior of IHA/TM, promoting the diffusion of Fe^2+^ and Mn^2+^ inside IHA/TM, and the adsorption rate is controlled by the diffusion rate inside the particles [41]. Based on the different slopes of the first and second stages, adsorption occurs in gradual stages, surface adsorption is controlled by the thickness of the boundary layer, and the diffusion rate constant *K*_2d_ is smaller than *K*_1d_ (Table 4), which indicates that the internal diffusion rate of the particles is slower. In addition, the curve of *q*_t_ versus *t*^1/2^ is bilinear over the entire time range, and the fitted curve does not cross the origin, indicating that the process of IHA/TM adsorption of Fe^2+^ and Mn^2+^ is not only controlled by intraparticle diffusion, but also affected by other adsorption stages [42,43].

### 3.7. Desorption and Reusability of IHA/TM

**Figure 8:**
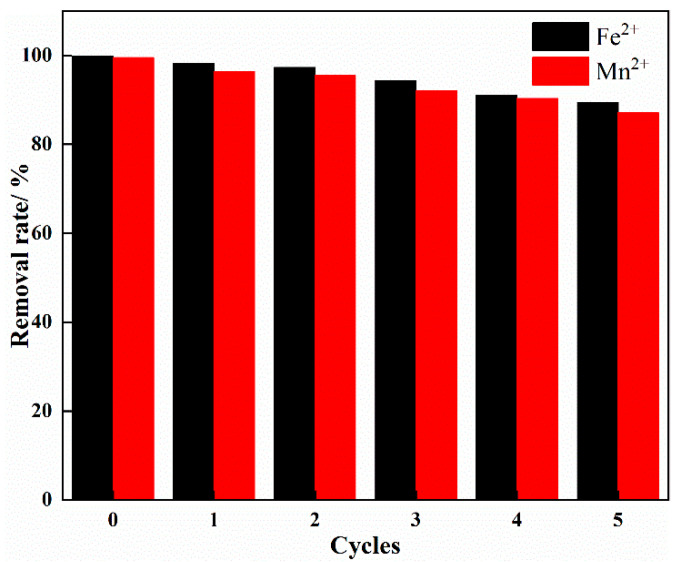
The influence of the number of adsorption-desorption cycles on the removal effect.

The results of the desorption experiment are given in Figure 8. Figure 8 shows that after 5 cycles, the removal rates of Fe^2+^ and Mn^2+^ decreased from 99.85% and 99.51% in the zeroth cycle to 89.4% and 87.07% in the fifth cycle (loss rates were 10.45% and 12.44%, respectively). The results showed that IHA/TM composite particles had good reusability and could remove Fe^2+^ and Mn^2+^ ions in actual groundwater.

### 3.8. Comparison of Saturated Adsorption Capacity of Materials

Table 5 shows that compared with similar adsorbents reported in the previous literature, the IHA/TM composite particles had certain advantages, including a relatively high saturated adsorption capacity for Fe^2+^ and Mn^2^; therefore, they are considered to be highly efficient adsorbents.

### 3.9. Microscopic Characterisation of IHA/TM Composite Particles

#### 3.9.1. XRD Analysis

**Figure 9:**
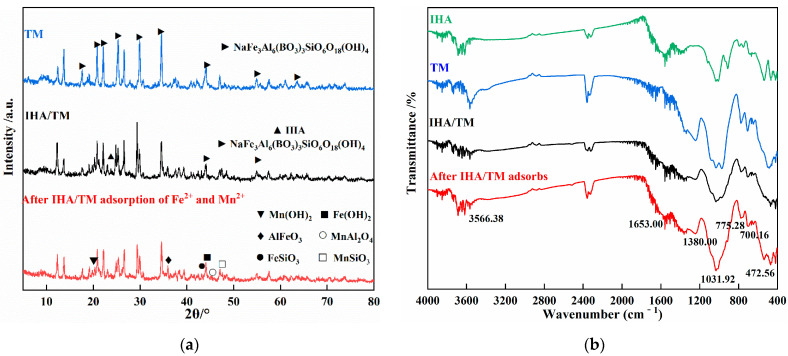
XRD pattern of IHA/TM before and after adsorption (**a**); FTIR spectra of IHA/TM before and after adsorption (**b**).

The crystal structure of the IHA/TM composite particles was studied by XRD, as shown in Figure 9a. TM is composed of NaFe_3_Al_6_(BO_3_)_3_SiO_6_O_18_(OH)_4_, which has several main characteristic peaks at 18.76°, 20.84°, 22.16°, 26.60°, 29.88°, 34.56°, 44.04°, 54.88° and 63.96°. All the characteristic peaks are consistent with the standard peaks of tourmaline reported in the literature [20]. By comparing the diffraction peaks of IHA/TM and raw materials, it was found that IHA/TM has diffraction peaks similar to those of TM, indicating that the sintering process does not destroy the crystal structures of the raw materials. After adsorption of Fe^2+^ and Mn^2+^, IHA/TM exhibited hydroxide precipitation containing manganese and iron, which reflected the electrode nature of TM. The adsorbent could react with Fe^2+^ and Mn^2+^ by chemical precipitation. Additionally, complex products containing manganese and iron also appeared, indicating that Fe^2+^ and Mn^2+^ underwent surface coordination reactions with the Al, Si, hydroxyl and carboxyl groups on the surface of the adsorbent.

#### 3.9.2. FTIR Analysis

Figure 9b shows the FTIR spectra of TM, IHA and IHA/TM. The absorption band at 472.56 cm^−1^ corresponds to Si-O, while the peaks at 700.16 cm^−1^ and 775.38 cm^−1^ are assigned to M-O (M=Fe or Al). The peaks at 1653 cm^−1^,1031.92 cm^−1^, 3500–3300 cm^−1^, 2950–2850 cm^−1^, 1720–1700 cm^−1^ and 1650–1580 cm^−1^ correspond to H-O-H, O-Si-O stretching, N-H stretching vibration peak and O-H stretching vibration, C-H aliphatic stretching, C=O stretching of the carboxyl group, and C=C stretching of the aromatic group, respectively [46]. It can be concluded from the above data that IHA contains numerous carboxyl and phenolic hydroxyl functional groups, which can be used as surface adsorption sites, so it has a good ability to adsorb heavy metals. Compared with those of IHA, the vibrations of the C=O and O-H stretching vibration peaks in the IHA/TM spectra were weaker, indicating that IHA may undergo dehydration and decarboxylation as the composite particles are heated during preparation, resulting in the loss of some acidic groups. However, it can be inferred from the spectra that the prepared composite particles retain the active functional groups of TM and IHA, so the preparation process does not affect the adsorption of metal ions.

After adsorption of Fe^2+^ and Mn^2+^ by IHA/TM, the functional groups changed significantly, and the original absorption band at 3566.38 cm^−1^ corresponds to the M-OH in TM [47]. After the reaction, the absorption band at 3566.38 cm^−1^ was significantly weakened, indicating that the hydroxyl M-OH on the TM surface combined with Fe^2+^ and Mn^2+^ in the solution to form surface complexes. The original O-H absorption band, N-H stretching vibration peak at 3500~3300 cm^−1^ and C=O absorption band at 1720–1700 cm^−1^ were significantly weakened and migrated, and the C-N absorption peak at 1380 cm^−1^ was shifted upwards. M. Adhikari also observed similar phenomena, which he believed were caused by the formation of complexes between metal ions and humic acid [48]. The change in functional groups indicated that N-H, C-N, carboxyl and hydroxyl groups were involved in the adsorption of Fe^2+^ and Mn^2+^ by IHA/TM.

#### 3.9.3. SEM Analysis

**Figure 10:**
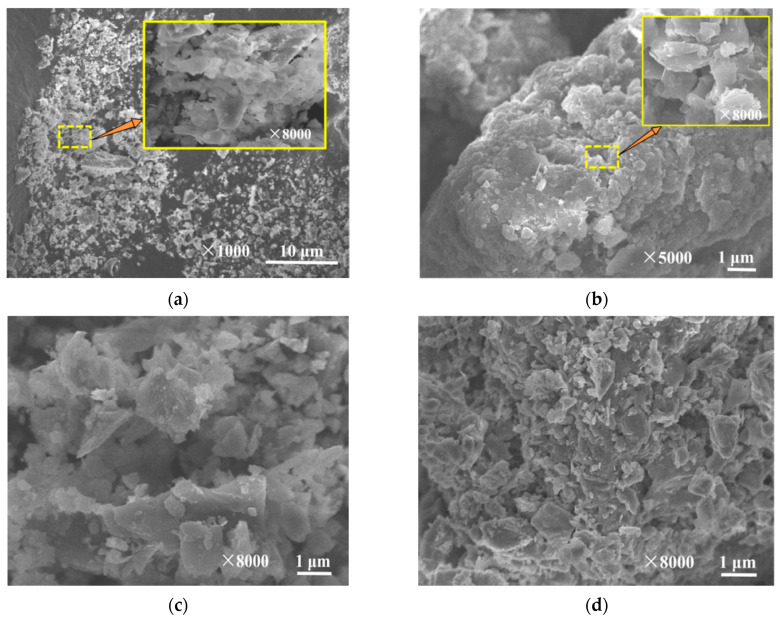
SEM images of IHA/TM before and after adsorption of Fe^2+^ and Mn^2+^: (**a**) TM; (**b**) IHA; (**c**) IHA/TM; (**d**) IHA/TM after adsorption of Fe^2+^ and Mn^2+^.

To further elucidate the morphological characteristics of IHA/TM, SEM images of TM, IHA and IHA/TM, before and after the reaction, were selected, as shown in Figure 10. As seen in Figure 10a, the surface of TM is in the form of a mutually stacked lamellar structure with a rough surface and cluster-like aggregation and adhesion properties, which is beneficial to the preparation of composite materials. As seen in Figure 10b, the surface of IHA is an inhomogeneous layered structure with an uneven surface that increases the specific surface area of the adsorbent and helps to adsorb heavy metal ions. Figure 10c shows that small tourmaline particles are attached to large humic acids, and there are flocculent or irregular flakes between the IHA surface and TM to combine the two, which may be because IHA reacts with Al, Si, Mg and Fe on TM to form IHA-A complexes (A represents Al, Si, Mg and Fe). After compounding IHA and TM, the voids and cavities on the surface of the insoluble IHA/TM composite particles increased, the adsorption sites increased, the specific surface area increased, the interparticle dispersion was more uniform, the particle shape was dense and irregular, the surface was much rougher and layered than the original TM, the polarity was reduced, the dispersion of TM was improved and the agglomeration problem of TM was solved. Figure 10d shows that after the adsorption of Fe^2+^ and Mn^2+^ by the IHA/TM composite particles, the surface structure morphology changed significantly, losing the complete cluster structure formed by large particles, forming small particles, appearing as a cover layer of particulate matter and producing a multitude of small particles and fragments because the surface of the IHA/TM adsorbent provides the necessary channels and sufficient adsorption space for the adsorption of Fe^2+^ and Mn^2+^ that facilitates the adsorption of Fe^2+^ and Mn^2+^. It is speculated that surface complexation may have occurred between the Fe^2+^ and Mn^2+^ ions and the IHA/TM composite adsorbent, and physical adsorption may have occurred at the surface. The soluble Fe^2+^, Mn^2+^, FeOH^+^, MnOH^+^, Fe(OH)_2(aq)_ and Mn(OH)_2(aq)_ were converted to Fe(OH)_2(s)_ and Mn(OH)_2(s)_.

#### 3.9.4. EDS Analysis

**Figure 11:**
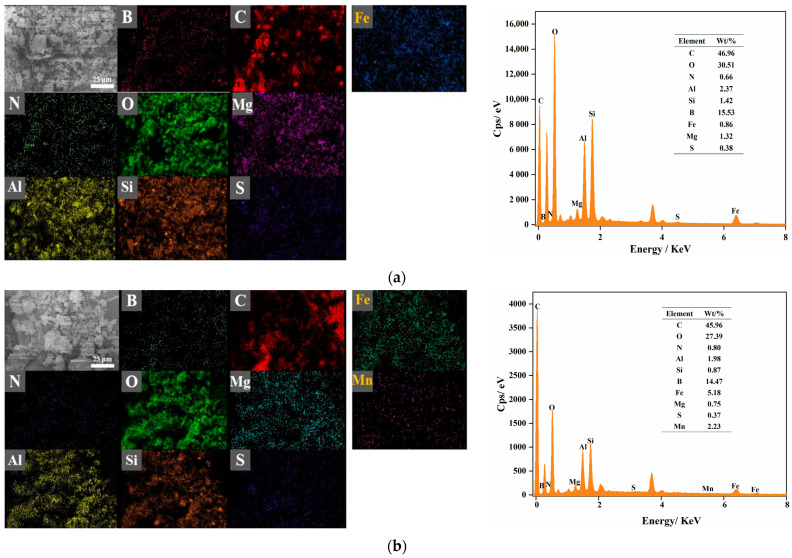
EDS surface scan images of IHA/TM composite particles before (**a**) and after (**b**) adsorption of Fe^2+^ and Mn^2+^.

The EDS spectra of the water samples before and after IHA/TM treatment are shown in Figure 11. Figure 11a shows that the main elements in IHA/TM composite particles were C, O, N, Al, Si, B and Fe, among which C, O, H and N were the main elements in IHA. After the adsorption of Fe^2+^ and Mn^2+^ by IHA/TM, the EDS (Figure 11b) spectrum showed higher levels of Fe than before the reaction, and a signal corresponding to Mn appeared at the same time, indicating that IHA/TM successfully adsorbed Fe^2+^ and Mn^2+^ ions in aqueous solution; in addition, the content of O, Al, Mg, Si and other elements decreased. It is speculated that the surface coordination reactions of Al, Si, hydroxyl and carboxyl groups with Fe^2+^ and Mn^2+^ ions may form surface complexes of Fe and Mn, resulting in a decrease in the O, Al and Si contents.

## 4. Mechanistic Analysis of Iron and Manganese Removal

The IHA/TM composite particles have good adsorption properties for Fe^2+^ and Mn^2+^, which is determined by the adsorption mechanism of TM and IHA. The structure of TM is compact and metal ions do not easily enter its crystal structure, so the adsorption of TM is mainly surface adsorption. TM is a cyclic silicate crystal mineral composed of elements such as Si, Al, Na, Ca, Mg, B, Fe, O and H. Its crystal structure can be regarded as composed of a [Si_6_O_18_] complex trigonal ring, [BO_3_] triangle and [Mg(Fe)-O_5_(OH)] triple octahedron with a common edge and a common vertex. There are many Si and Al elements on the surface of the mineral. It easily reacts with water in aqueous solution to form a hydroxylated surface (≡MeOH) (chemical Equations (15) and (16)). Chemical Equation (15) shows that the Si-O bond within the Si_6_O_18_ six-membered ring breaks and interacts with water molecules, with the Si bond bonding to OH and the O bonding to H, resulting in a bonded hydroxyl group on the surface of TM. Chemical Equation (16) shows that the intraoctahedral bond of AlO_6_ is also broken, and then the hydroxyl group is bonded, resulting in the exposure of a large number of metal cations. Then it forms surface complexes with Fe^2+^ and Mn^2+^ in water (chemical Equations (17) and (18)). Since Fe^2+^ and Mn^2+^ have empty orbitals, they tend to form ligands [49]. At the same time, the molecular formula of IHA is C_9_H_9_NO_6_, the basic structural unit is an aromatic ring, and the alkyl chain contains a variety of functional groups such as -NH_2_, -COOH, -OH and -C=O. These groups can be closely combined with metals, giving IHA a high adsorption capacity for metals. Therefore, Fe^2+^ and Mn^2+^ can be coordinated and adsorbed on the surface of IHA/TM to form complexes and generate chemical bond forces, which also confirms the conclusion of the adsorption kinetics. Additionally, the variation in surface functional groups in FTIR indicates that functional groups such as M-OH, NH, CN, carboxyl and hydroxyl groups are involved in the surface complexation reaction of IHA/TM on Fe and Mn ions. This result is consistent with the complexation products of AlFeO_3_, FeSiO_3_, MnAl_2_O_4_ and MnSiO_3_ obtained by XRD.

(15) $${\;\mathrm{-Si}+\mathrm{OH}}^{-}{=\mathrm{-SiOH};\;\mathrm{-SiO}+H}^{+}=\mathrm{-SiOH};$$

(16) $${\mathrm{-Al}+\mathrm{OH}}^{-}{=\mathrm{-AlOH};\;\mathrm{-AlO}+H}^{+}=\mathrm{-AlOH};$$

(17) $$\equiv \mathrm{MeOH}+{\mathrm{Fe}}^{2+}\to \equiv {\mathrm{MeOFe}}^{+}+H^{+}$$

(18) $$\equiv \mathrm{MeOH}+{\mathrm{Mn}}^{2+}\to \equiv {\mathrm{MeOMn}}^{+}+H^{+}$$

**Figure 12:**
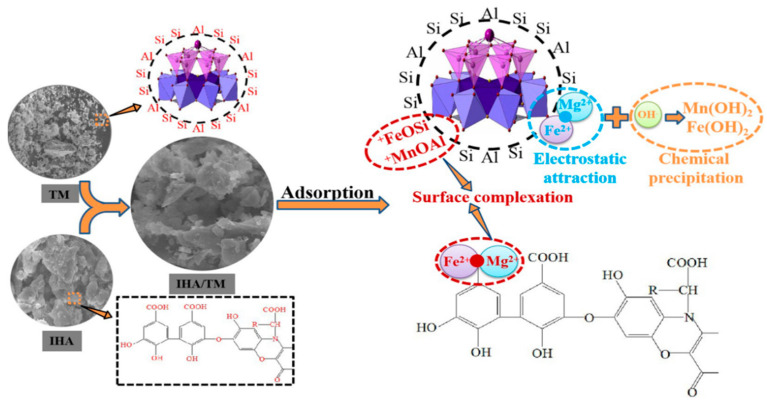
Schematic diagram of the adsorption mechanism of iron and manganese by IHA/TM composite particles.

In addition, based on the electrode nature of TM in IHA/TM, under the action of an electric field, the negative electrode of TM particles adsorbs Fe^2+^ and Mn^2+^ to its surface through electrostatic gravity, resulting in an increase in the concentration of Fe^2+^ and Mn^2+^ around the negative electrode, which combines with OH^−^ in water and reaches a certain concentration to produce precipitation (chemical Equations (19) and (20)), thus decreasing the solution of Fe^2+^ and Mn^2+^ concentration. The positive electrode of TM particles can adsorb OH^−^ in water, leading to an increase in the concentration of OH^−^ around it, again favouring the aggregation of ions in solution and the formation of insoluble precipitates [50]. This is consistent with the detection of Fe(OH)_2_ and Mn(OH)_2_ in the XRD analysis. The removal mechanism of IHA/TM is shown in Figure 12.

(19) $${2\mathrm{OH}}^{-}+{\mathrm{Fe}}^{2+}\to {\mathrm{Fe}(\mathrm{OH})}_{2}↓$$

(20) $${2\mathrm{OH}}^{-}+{\mathrm{Mn}}^{2+}\to {\mathrm{Mn}(\mathrm{OH})}_{2}↓$$

## 5. Conclusions

(1) Using the respective advantages of IHA and TM, IHA/TM composite particles were made to study the effect of adsorbing the heavy metals iron and manganese ions from polluted groundwater. The results showed that IHA/TM composite particles can effectively remove iron and manganese ions from groundwater. The optimal adsorption conditions of IHA/TM for Fe^2+^ and Mn^2+^ were as follows: TM:IHA was 2:3, the dosage of IHA/TM was 10 g/L, the pH was 6, the reaction time was 240 min, and the temperature was 35 °C. The equilibrium adsorption capacities of Fe^2+^ and Mn^2+^ were 5.645 mg/g and 3.574 mg/g, respectively. After five cycles of regeneration, the rates of Fe^2+^ and Mn^2+^ removal decreased by 10.45% and 12.44%, respectively. IHA/TM has good reusability and great potential for removing metals from water.

(2) The process of Fe^2+^ and Mn^2+^ adsorption onto IHA/TM conformed to the pseudosecond order kinetic model and Langmuir equation (*R*^2^ > 0.99). The experimental thermodynamic results were Δ*G* < 0, Δ*H* > 0 and Δ*S* > 0, indicating that the adsorption of Fe^2+^ and Mn^2+^ onto TM tended to be monolayer adsorption, and the adsorption process was spontaneous, endothermic and sustainable.

(3) Through XRD, FTIR, SEM and EDS analysis, it was confirmed that surface coordination and chemical precipitation are the main mechanisms of Fe^2+^ and Mn^2+^ removal by IHA/TM.

(4) The study is a laboratory simulation of high-concentration Fe^2+^ and Mn^2+^ polluted water, and no actual water samples are used for research. In the future, actual water samples in the field should be used to further study the influence of other coexisting ions in the polluted water on the adsorption of Fe^2+^ and Mn^2+^ by IHA/TM composite particles, and dynamic experimental research carried out in order to create conditions for the application of IHA/TM adsorbents in practical engineering.

## Figures and Tables

**Table 1:** Fitting results of the isothermal model for IHA/TM adsorption of iron and manganese.

Ions Type	Temperature (°C)	Langmuir			Freundlich			Temkin		
		*q*_m_ mg/g	*K*_L_	*R* ^2^	*K*_F_	1/n	*R* ^2^	ln*A*	*B*	*R* ^2^
Fe^2+^	25	5.010	0.399	0.992	2.402	0.189	0.982	3.145	0.684	0.978
	35	5.175	0.459	0.991	2.597	0.172	0.983	3.635	0.663	0.970
	45	5.319	0.525	0.991	2.828	0.157	0.988	4.374	0.621	0.963
Mn^2+^	25	3.465	0.534	0.992	1.556	0.224	0.900	3.772	0.449	0.964
	35	3.596	0.640	0.990	1.861	0.181	0.951	5.594	0.370	0.972
	45	3.659	0.685	0.990	2.054	0.155	0.953	7.169	0.318	0.960

**Table 2:** Thermodynamic parameters of IHA/TM adsorption of Fe^2+^ and Mn^2+^.

Ion Type	T (°C)	*C*_e_ (mg/L)	*q*_e_ (mg/g)	Δ*G* (kJ/mol)	Δ*H* (kJ/mol)	Δ*S* (J/(mol k))
Fe^2+^	25	0.54	2.46	−3.74	33.64	138.24
	35	0.47	2.47	−4.12		
	45	0.27	2.58	−5.95		
Mn^2+^	25	0.39	0.97	−2.25	22.15	86.58
	35	0.24	0.99	−3.61		
	45	0.23	1.00	−3.89		

**Table 3:** Pseudo-first order and pseudo-second order kinetic fitting results.

Ions Type	Concentration mg/L	Quasi-First Order Dynamics			Quasi-Second Order Dynamics		
		*q*_e_ mg/g	*K*_1_ 1/min	*R* ^2^	*q*_e_ mg/g	*K*_2_ mg/g/min	*R* ^2^
Fe^2+^	25	2.119	0.0203	0.889	2.837	0.0131	0.996
	40	2.973	0.0303	0.965	2.462	0.0132	0.993
	60	5.159	0.0243	0.942	4.939	0.0051	0.997
	80	6.912	0.0311	0.921	6.003	0.0030	0.995
	100	7.853	0.0264	0.921	7.412	0.0021	0.991
Mn^2+^	10	0.541	0.0178	0.959	1.052	0.0602	0.999
	20	0.924	0.0120	0.995	2.035	0.0255	0.996
	30	1.543	0.0125	0.991	2.721	0.0131	0.994
	40	2.445	0.0122	0.975	3.205	0.0085	0.995
	50	3.188	0.0140	0.986	3.451	0.0042	0.999

**Table 4:** Internal diffusion model fitting results.

Ion Type	Concentration mg/L	*K*_1d_ mg/g·min^−1/2^	*R*_1_^2^	*K*_2d_ mg/g·min^−1/2^	*R*_2_^2^
Fe^2+^	25	0.088	0.734	0.076	0.952
	40	0.196	0.978	0.018	0.637
	60	0.317	0.925	0.038	0.916
	80	0.372	0.974	0.066	0.674
	100	0.483	0.970	0.039	0.664
Mn^2+^	10	0.032	0.991	0.024	0.950
	20	0.073	0.962	0.051	0.972
	30	0.117	0.914	0.079	0.972
	40	0.135	0.815	0.126	0.983
	50	0.270	0.991	0.135	0.997

**Table 5:** Comparison of the IHA/TM composite adsorbent with previously reported adsorbents for the removal of iron and manganese in terms of uptake capacity.

Adsorbent	pH	*q*_m_ of Fe^2+^(mg/g)	*q*_m_ of Mn^2+^(mg/g)	Reference
Sugarcane bagasse	4.5		0.676	[44]
Graptolite	6	0.352		[45]
Limestone	6.2–6.7	0.03	0.007	[2]
Granular activated carbon	7	3.601	2.545	[6]
Slovakian natural zeolite	7	1.157	0.075	[7]
Natural shells	7.0–9.0	4.00	3.50	[8]
IHA/TM composite granules	6.0	5.645	3.574	This study

## Data Availability

Data can be obtained from corresponding authors upon reasonable request.
